# Whole-transcriptome sequencing reveals the effects of acupuncture on early embryos post-IVF-ET in poor ovarian response

**DOI:** 10.1186/s13048-025-01682-7

**Published:** 2025-04-30

**Authors:** Jianheng Hao, Yuemeng Zhao, Xuan Liu, Huichao Xu, Liying Liu, Haijun Wang, Ying Lan, Laixi Ji

**Affiliations:** 1 School of Acupuncture-Moxibustion and Tuina, Chengdu University of Traditional Chinese Medicine, Chengdu, 610075 Sichuan China; 2 Chengdu University of Traditional Chinese Medicine Affiliated Hospital, Chengdu, 610075 Sichuan China; 3 The Second Clinical College, Shanxi University of Chinese Medicine, Jinzhong, 030619 Shanxi China

**Keywords:** Acupuncture, Poor ovarian response, Whole-transcriptome sequencing, In vitro fertilization-embryo transfer, Differentially expressed genes

## Abstract

**Background:**

Poor ovarian response (POR) refers to a pathological condition where the ovaries respond inadequately to gonadotropin stimulation during ovulation induction. The decline in both the quantity and quality of available oocytes makes achieving pregnancy through IVF-ET more challenging. Several studies have demonstrated that acupuncture can improve embryo quality, increase the number of high-quality embryos, and enhance pregnancy outcomes in IVF-ET patients, although the underlying mechanisms remain unclear. Therefore, this study aimed to investigate the molecular mechanisms by which acupuncture influences early embryo development in POR mice after IVF-ET through whole-transcriptome sequencing.

**Methods:**

We established POR mice model and performed acupuncture treatment. Fresh denuded oocytes were retrieved and transplanted via IVF-ET into new donor female mice to obtain early-stage embryo tissues. Vaginal smear tests were conducted to monitor estrous cycle changes, while oocyte retrieval, ovarian wet weight, and ovarian index were assessed. Serum concentrations of anti-Müllerian hormone (AMH), follicle-stimulating hormone (FSH), estradiol (E2), and luteinizing hormone (LH) were measured via ELISA. Histopathological changes and apoptosis in the ovaries were evaluated using HE and TUNEL staining. Whole-transcriptome sequencing was employed to establish expression profiles of differentially expressed mRNAs (DEmRNAs), DEmiRNAs, DElncRNAs, and DEcircRNAs. The circ/lncRNA-miRNA-mRNA network was constructed to analyze the biological functions and potential mechanisms of acupuncture on early embryo development post-IVF-ET in POR mice.

**Results:**

Our results demonstrated that acupuncture improved ovarian function in POR mice, corrected serum hormone imbalances, and alleviated abnormal apoptosis in ovarian granulosa cells. Through whole-transcriptome sequencing, we identified 685 DEmRNAs, 13 DEmiRNAs, 325 DElncRNAs, and 4 DEcircRNAs exhibiting regulatory trends. By constructing the circ/lncRNA-miRNA-mRNA network, we found that miR-291a-3p, miR-294-3p, and miR-295-3p may serve as key targets influencing early embryo development via multiple pathways, including the Toll-like receptor signaling pathway and p53 signaling pathway.

**Conclusion:**

Overall, our study is the first to reveal the molecular mechanisms by which acupuncture improves early embryo development under POR conditions, providing new evidence for the use of acupuncture in assisted reproduction.

**Supplementary Information:**

The online version contains supplementary material available at 10.1186/s13048-025-01682-7.

## Introduction

With advancements in reproductive medicine, assisted reproductive technologies (ART) such as in vitro fertilization-embryo transfer (IVF-ET) have become essential for many infertile patients to achieve their reproductive goals. However, poor ovarian response (POR) remains a significant clinical challenge, not only affecting the success rate of IVF-ET but also significantly increasing the risk of pregnancy complications [1–2]. POR refers to the inadequate ovarian response to gonadotropin stimulation during ovarian stimulation treatment, characterized by a reduced number of mature oocytes, poor follicle development, and low serum estradiol levels. These factors ultimately lead to a decrease in the number of high-quality embryos, lower embryo implantation success rates, and subsequently impact the likelihood of achieving pregnancy [2–3]. The etiology of POR is complex and not fully understood, but it is associated with aging, genetic factors, diminished ovarian reserve, environmental factors, and autoimmune disorders. As women age, the ovarian follicle pool is progressively depleted, leading to a decline in ovarian reserve and a reduced response to stimulation drugs [4]. Additionally, polymorphisms in genes such as FSHR and AMHR2 are closely linked to POR [5–6]. Research also suggests that oxidative stress and mitochondrial dysfunction in the ovarian microenvironment may play a crucial role in the development of POR [7]. Despite ongoing efforts to optimize treatment strategies for POR, significant limitations remain [8–9]. Increasing the dose of gonadotropins often fails to substantially improve ovarian response and may lead to ovarian hyperstimulation syndrome (OHSS). While individualized treatment approaches can be tailored to the patient’s ovarian reserve, their efficacy varies, and some patients still struggle to produce a sufficient number of high-quality oocytes. Therefore, existing treatment options for enhancing IVF-ET success rates in POR patients are still limited, highlighting the need for further breakthroughs in overcoming these challenges.

Although IVF-ET has brought hope to infertile patients, concerns remain regarding its low clinical pregnancy rates and potential risks to offspring. Epidemiological studies indicate that the risks associated with ART are higher than those of natural conception [10–11]. Consequently, recent research has increasingly focused on improving the quality of embryos supported by IVF-ET and addressing issues such as birth defects in offspring. The quality of early embryos is crucial for the pregnancy outcomes and health of offspring following IVF-ET. High-quality early embryos can develop into healthy fetuses, while poor-quality embryos may carry chromosomal abnormalities or genetic mutations, leading to developmental arrest or birth defects [12]. Therefore, assessing early embryo quality during IVF-ET is of great importance. POR presents a significant challenge for achieving pregnancy via IVF-ET, as the limited availability of oocytes reduces the likelihood of obtaining high-quality embryos for transfer, thereby decreasing implantation and pregnancy success rates. Recent evidence suggests that acupuncture may have positive effects on IVF-ET outcomes in POR patients by regulating hormonal balance and improving ovarian function, potentially enhancing embryo quality and increasing the number of high-quality embryos, thereby improving pregnancy outcomes [13–15]. However, current research primarily focuses on the effects of acupuncture on maternal ovarian function, while in-depth exploration of embryos formed from oocytes treated with acupuncture and subjected to IVF-ET remains limited. Whole-transcriptome sequencing is a high-throughput technology capable of comprehensively analyzing all types of transcripts within a biological system, including messenger RNAs (mRNAs) and various non-coding RNAs (ncRNAs) such as microRNAs (miRNAs), circular RNAs (circRNAs), and long non-coding RNAs (lncRNAs) [16]. By quantifying and analyzing the expression levels and types of these RNA molecules, whole-transcriptome sequencing not only helps us understand the developmental abnormalities in POR embryos, such as decreased embryo quality and reduced numbers of high-quality embryos, but also elucidates the changes in gene expression before and after acupuncture intervention, thereby revealing the molecular mechanisms by which acupuncture improves embryo development. Therefore, this study, based on whole-transcriptome sequencing, reveals the effects of acupuncture on early embryos in POR mice following IVF-ET, aiming to provide a meaningful research foundation for the application of acupuncture in ART.

## Materials and methods

### Experimental animals

SPF-grade female and male C57BL/6 mice, aged 7–8 weeks, were purchased from Beijing Sibeifu Company. The body weight of female mice was 17 ± 1 g, and that of male mice was 19 ± 1 g. The license number for the production of experimental animals is SCXK (Beijing) 2019-0010. The mice were housed in the animal experiment research center at Shanxi University of Chinese Medicine, under dry and ventilated conditions with a 12-hour light/dark cycle, a temperature range of 23–25℃, and humidity levels of 45-55%. The mice had free access to food and water. All animal experiments adhered to NIH guidelines and received approval from the experimental animal ethics committee of Shanxi University of Chinese Medicine (approval No. AWE202303382).

### Grouping and modeling

**Fig. 1:**
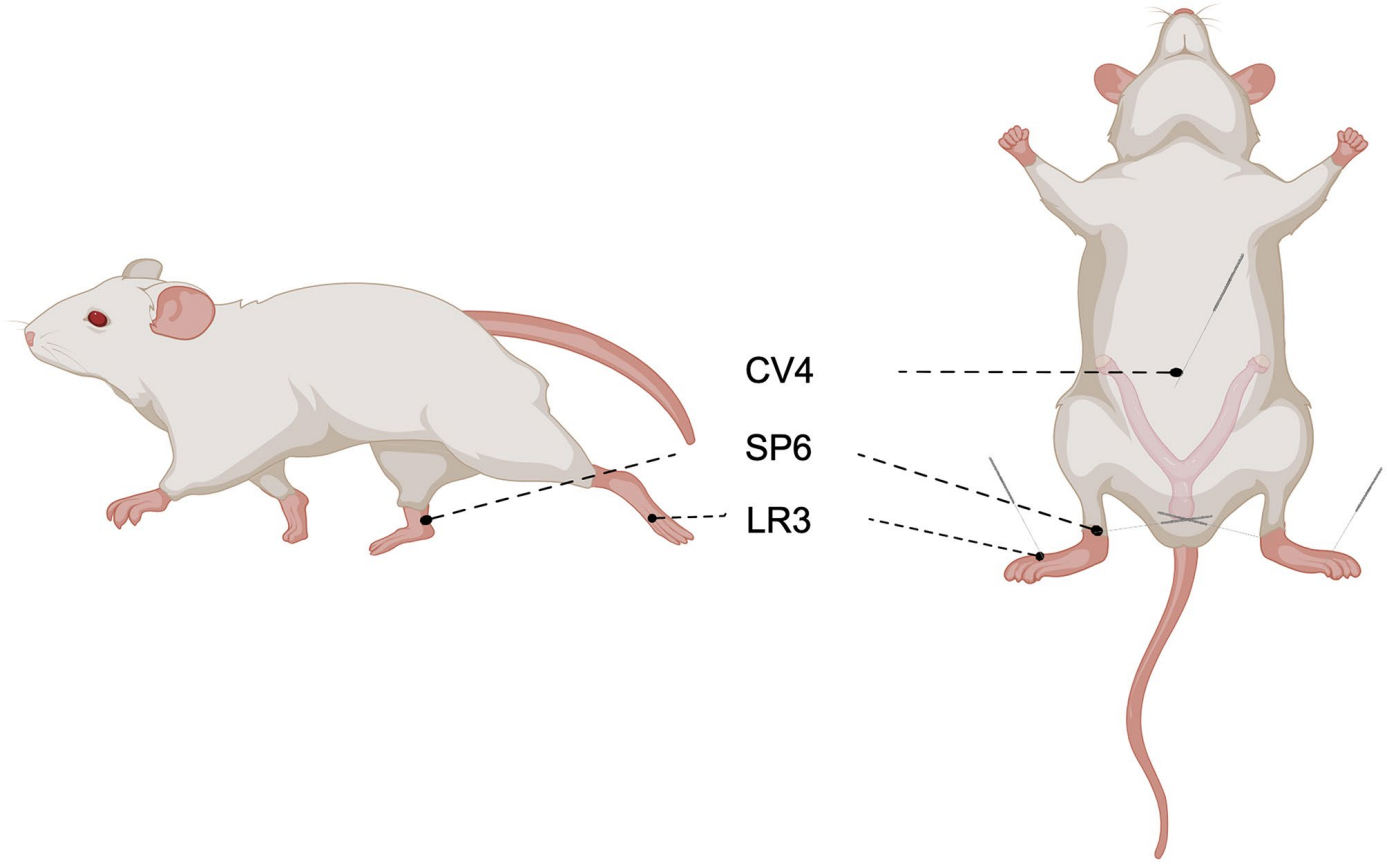
Schematic diagram of acupuncture points

Thirty female mice with normal estrous cycles were randomly divided into three groups: control group, model group, and acupuncture (ACU) group (*n* = 10). Each morning, the model and ACU groups received an intragastric administration of a suspension of tripterygium glycosidesto (Z43020138, Qianjin Xieli Pharmaceutical Co., Ltd., Hunan, China) induce the POR mice model (50 mg·kg^− 1^), while the control group received an equal volume of 0.9% sodium chloride solution for 14 days [17]. Starting from day 15, the ACU group mice were subjected to acupuncture intervention every morning at 10:00 AM. During the experiment, the mice were fixed in a specialized restrainer, maintaining a supine position to ensure stability and minimize unnecessary stress. Prior to acupuncture, the acupuncture sites were disinfected with 75% ethanol. Acupuncture was performed using disposable sterile needles (0.18 mm × 13 mm, Huatuo Medical Equipment Co., Ltd., Suzhou, China), and the selected acupuncture points included Taichong (LR3, bilateral), Sanyinjiao (SP6, bilateral), and Guanyuan (CV4). Specifically, Taichong (LR3) was located on the hind paw, approximately 1/3 from the forefoot, Sanyinjiao (SP6) was located approximately 5 mm above the medial malleolus of the hind leg, and Guanyuan (CV4) was located on the midline of the abdomen, dividing the distance between the pubic symphysis and the xiphoid process into 17 equal parts, with Guanyuan (CV4) located 2/17 upwards from the pubic symphysis (Fig. 1). During the procedure, the Guanyuan (CV4) point was needled obliquely towards the ovaries, with a needle depth of about 5 mm to stimulate the abdominal region; while the Taichong (LR3, bilateral) and Sanyinjiao (SP6, bilateral) points were needled perpendicularly with a depth of about 2 mm, the needles being inserted vertically into the subcutaneous tissue. Each acupuncture session lasted for 20 min, without any manipulation or electroacupuncture stimulation during needle retention. At the same time, the experimenters observed the condition of the mice to ensure the safety and effectiveness of the acupuncture process. After each session, the needles were carefully removed, and the acupuncture points were disinfected again to prevent infection. The acupuncture treatment was performed once daily for 14 consecutive days. Additionally, to control for the stress induced by restraint, at the same time as acupuncture intervention, the mice in the control and model groups were placed in the restrainer for 20 min daily without any acupuncture treatment. The day after the treatment ended, superovulation was induced by intraperitoneal injection of 5 IU of Pregnant Mare Serum Gonadotropin (PMSG, M2620, Aibei Biotechnology Co., Ltd, Nanjing, China) in all groups of female mice, followed by an injection of 5 IU of Human Chorionic Gonadotropin (HCG, M2520, Aibei Biotechnology Co., Ltd, Nanjing, China) 48 h later. Fourteen hours after HCG injection, the mice were anesthetized with isoflurane, and samples were collected according to experimental requirements for subsequent analysis.

### IVF-ET

#### Oocyte retrieval

Ten female mice from each group were anesthetized with isoflurane gas, and the oviducts were excised and placed in a dish. The ampulla of the oviduct was carefully torn open using fine forceps, and gentle pressure was applied to release the oocyte-cumulus complex. The complex was then transferred to M2 medium (M1250, Aibei Biotechnology, Nanjing, China) for 5 min. To obtain denuded oocytes, cumulus cells were removed by pipetting with a fine glass pipette of similar diameter to the oocytes.

#### Sperm retrieval

Fifteen male mice that had not mated within the past week were selected. After being anesthetized with isoflurane gas (R510-22-10, Shenzhen RWD Life Science Co., Ltd, China), the abdomen was opened to extract the epididymis and vas deferens, which were placed in a pre-equilibrated sperm capacitation dish. The tail of the epididymis was cut open and gently squeezed with ophthalmic forceps, allowing sperm to enter the HTF medium drop (M1135, Aibei Biotechnology Co., Ltd, Nanjing, China). After removing the epididymal tissue, the sample was incubated for 10 min. Once sperm dispersion was observed under a microscope, the sample was incubated at 37 °C in a 5% CO_2_ incubator (BL-J80-S, Shanghai Boxun Industrial Co., Ltd) for 50 min before use.

#### IVF

Freshly retrieved denuded oocytes and capacitated sperm were added to pre-equilibrated HTF fertilization dishes and incubated for 6 h. The fertilized eggs were then transferred to pre-equilibrated KSOM medium drops (M1435, Aibei Biotechnology Co., Ltd, Nanjing, China) and repeatedly pipetted to remove residual sperm from the zona pellucida. The embryos were then transferred to a new medium drop and cultured for 4 h until reaching the 2-cell stage.

#### Vasectomy in male mice

One week before the start of the embryo transfer, another 15 male mice underwent vasectomy. After being anesthetized with isoflurane, the mice were fixed, and the abdomen was sterilized. The abdominal cavity was opened, and the vas deferens were cauterized using ophthalmic forceps heated over an alcohol lamp flame. The incision was then sutured. The mice were placed in a warm area until recovery and then returned to clean cages with sterile bedding.

#### Preparation of pseudopregnant female mice

An additional 30 female mice were paired with vasectomized males in a 2:1 ratio. The next day, vaginal plugs were checked, and the presence of a plug was considered as 0.5 days of pseudopregnancy.

#### ET and sample collection

Pseudopregnant female mice at 1.5 days were anesthetized with isoflurane gas, and their backs were sterilized before making a dorsal incision. Two-cell stage embryos from each group were gently transferred into the ampulla of both oviducts using a transfer needle (H0450, Aibei Biotechnology Co., Ltd, Nanjing, China), with five embryos per side. The incision was sutured after transfer. On the 7th day post-transfer, the mice were euthanized by cervical dislocation under isoflurane anesthesia. The abdominal cavity was opened to observe uterine pregnancy status and count the number of embryos. Implantation sites were collected and stored at -80 °C for further analysis.

### Estrous cycle observation

The tail of the mice were gently restrained to fully expose the vaginal opening. A small cotton swab dipped in physiological saline was inserted approximately 0.3 cm into the mouse’s vagina and gently rotated. The swab was then evenly smeared onto a glass slide and subjected to routine HE staining (DH0006, Leagene, Beijing, China). The changes in the estrous cycle were observed under a light microscope (ECLIPSE Ci-S, Nikon, Tokyo, Japan).

### Detection of oocyte count, ovarian wet weight, and ovarian index

Both ovaries were aseptically excised from the mice, and excess adipose tissue was removed. The ovaries were placed on filter paper to absorb excess moisture, and their wet weight was measured using an electronic balance. The ovarian index was calculated as the ratio of the wet weight of both ovaries to the final body weight recorded before euthanasia. Additionally, the number of denuded oocytes was observed and counted under a microscope.

### Determination of serum AMH, FSH, LH, and E2 concentrations

Serum concentrations of anti-Müllerian hormone (AMH), follicle-stimulating hormone (FSH), luteinizing hormone (LH), and estradiol (E2) were measured using ELISA kits (MU30265, MU30399, MU30382, MU30395; all purchased from Bio-swamp, Wuhan, China) following the manufacturer’s instructions. A total of 50 µL of standards and samples were added to each well of a 96-well plate and incubated at 37 °C for 90 min. After discarding the liquid, 100 µL of biotinylated antibody working solution was added to each well, and the plate was incubated at 37 °C for 1 h. The plate was then washed and dried, and 100 µL of enzyme conjugate working solution was added to each well, followed by incubation at 37 °C for 30 min. After washing and drying the plate again, 90 µL of substrate solution was added to each well, and the plate was incubated at 37 °C for 15 min. Finally, 50 µL of stop solution was added to each well to terminate the reaction, and the absorbance at 450 nm was measured using a microplate reader. A standard curve was plotted, and the concentrations of AMH, FSH, LH, and E2 were calculated accordingly.

### Hematoxylin and Eosin (H&E) staining

Ovarian tissues from the mice were fixed in 4% paraformaldehyde for 48 h, followed by dehydration, paraffin embedding, sectioning, hematoxylin and eosin staining, dehydration, and mounting. The pathological changes in the ovarian tissues were then observed and analyzed under a light microscope.

### Transmission electron microscopy (TEM) detection

Fresh ovarian tissue samples were collected and rinsed with electron microscopy buffer. The tissues were fixed in glutaraldehyde at room temperature for 2 h, followed by rinsing and post-fixation with osmium tetroxide. After gradient dehydration, embedding, polymerization, sectioning, and staining, the samples were examined, and images were captured using a transmission electron microscope.

### TUNEL staining

Ovarian paraffin sections were deparaffinized and rehydrated, followed by washing with PBS three times for 5 min each. The TUNEL assay kit (G1504, Servicebio, Wuhan, China) was utilized, with an appropriate amount of TDT enzyme and dUTP added, and the samples were incubated at 37 °C for 2 h. After incubation, the samples were washed with PBS three times for 5 min each. DAPI was then added, and the samples were incubated at room temperature for 10 min. Following another round of PBS washes, the sections were mounted with an anti-fade aqueous mounting medium. Finally, images were captured and analyzed using a fluorescence microscope (DM750, LEICA, Wetzlar, Germany).

### Western blot analysis

Total protein was extracted from mouse ovarian tissue, and protein concentration was determined using the BCA assay. Samples were subjected to electrophoresis (130 V constant voltage, 40 min), followed by wet transfer to membranes (400 mA constant current, 30 min). After blocking for 2 h, the membranes were incubated overnight at 4 °C with antibodies against B-cell lymphoma-2 (BCL-2), BCL-2-associated X protein (BAX), and Caspase-3 (GB124830, GB12690, GB11767C, all from Servicebio, Wuhan, China), with dilutions of 1:1000 for BAX, BCL-2, and Caspase-3, and 1:3000 for β-actin. The next day, the membranes were washed and incubated with the appropriate secondary antibody for 1 h (1:5000 dilution), followed by the addition of chemiluminescent substrate. The membranes were then exposed using a gel imaging system (HOOD-II, Bio-Rad, California, USA). Image grayscale values were analyzed using Image J, and the relative protein expression levels were calculated by comparing the grayscale values of target proteins to that of the internal control.

### Whole transcriptome sequencing and analysis

#### RNA extraction and detection

Three embryo tissue samples were randomly selected from each group, and total RNA was extracted using the TRIzol method. Approximately 50 mg of embryo tissue was homogenized using a grinder, followed by the addition of TRIzol reagent (15596018, Invitrogen, Thermo Fisher Scientific, Massachusetts, USA) and thorough mixing. After incubation for 5 min, the sample was centrifuged at 12,000 rpm for 5 min, and the supernatant was collected. Chloroform (10006818, Sinopharm Chemical Reagent, Shanghai, China) was then added, vigorously shaken, incubated for 2–3 min, and centrifuged at 12,000 rpm for 15 min at 4 °C. The colorless aqueous phase was carefully collected and mixed with isopropanol (80109218, Sinopharm Chemical Reagent, Shanghai, China). After incubation for 10 min, the sample was centrifuged at 12,000 rpm for 10 min at 4 °C. The supernatant was discarded, and the RNA pellet was resuspended in 75% ethanol, followed by centrifugation at 12,000 rpm for 5 min at 4 °C. The ethanol was then removed, and the RNA was dissolved in RNase-free water. The RNA concentration and purity were measured using a spectrophotometer (NanoDrop 2000, Thermo Scientific, Waltham, Massachusetts, USA), while RNA integrity was assessed using an Agilent Bioanalyzer (Agilent 2100, Agilent, California, USA).

#### The library construction process for mRNA, lncRNA and circRNA

For the total RNA, the Epicentre Ribo-Zero™ rRNA Removal Kit (15066012, Illumina, California, USA) was used to remove ribosomal RNA from the total RNA. Subsequently, the RNA was randomly fragmented by divalent cations through an ion fragmentation method. Using the RNA as a template and random oligonucleotides as primers, the first strand of cDNA was synthesized. Then, the RNA strand was degraded by RNaseH, and under the DNA polymerase I system, dNTPs in which dUTP was substituted for dTTP were used as raw materials to synthesize the second strand of cDNA. The double-stranded cDNA was purified, and then both ends were repaired, an “A” base was introduced at the 3’ end, and the sequencing adaptor was ligated. At this time, USER enzyme (M5509, NEB, Massachusetts, USA) was added to degrade the second strand of cDNA containing U. cDNA fragments of approximately 400–500 bp were screened using AMPure XP beads (A63880, Beckman Coulter, California, USA), followed by PCR amplification. The PCR products were purified again using AMPure XP beads, and finally, the library was obtained. The quality of the library was detected using the Agilent Bioanalyzer (2100, Agilent, California, USA) and the Agilent High Sensitivity DNA Kit (5067 − 4626, Agilent, California, USA). The total library concentration was detected by the PicoGreen assay (Quantifluor-ST fluorometer, E6090, Promega, Wisconsin, USA; Quant-iT PicoGreen dsDNA Assay Kit, P7589, Invitrogen, California, USA), and the effective library concentration was quantitatively detected by qPCR (StepOnePlus Real-Time PCR Systems, Thermo Scientific, Waltham, Massachusetts, USA). The multiplexed DNA libraries were normalized and then mixed in equal volumes. After the mixed library was gradually diluted and quantified, sequencing was performed on an Illumina sequencer in the PE150 mode (This part of the work was assisted by Shanghai Personal Biotechnology Co., Ltd., China).

#### The library construction process for miRNA

The total RNA was used to construct the library with the NEB Next Multiplex Small RNA Library Prep Set for Illumina (E7300, New England Biolabs, Ipswich, Massachusetts, USA). The 3’ and 5’ adaptors were ligated using ligases. Then, the RNA was reverse-transcribed into double-stranded cDNA with Superscript II reverse transcriptase (18064014, Invitrogen, Thermo Fisher Scientific, Massachusetts, USA). PCR amplification was carried out to enrich the DNA fragments. According to the fragment size, the products of the target fragment size were separated using a 15% PAGE gel, and finally, the library was obtained. The quality of the library was detected using the Agilent 2100 Bioanalyzer and the Agilent High Sensitivity DNA Kit. The total library concentration was detected by the PicoGreen assay, and the effective library concentration was quantitatively detected by qPCR. The multiplexed DNA libraries were normalized and then mixed in equal volumes. After the mixed library was gradually diluted and quantified, sequencing was performed on an Illumina sequencer in the PE150 mode.

#### Analysis method

##### Data quality control, filtering, and mapping analysis

The quality of the raw data in FASTQ format was first assessed, and then filtered using fastp (v0.22.0) software. Clean data were obtained by removing reads containing adapters, poly-N, and low-quality reads. All subsequent analyses were conducted using high-quality clean data. The reference genome and gene annotation files were downloaded from a genome website, with Mus_musculus.GRCm39.dna.primary_assembly.fa (GCA_000001635.9) used as the reference genome. The filtered reads were mapped to the reference genome using HISAT2 (v2.0.5). The alignment region distribution of the mapped reads was then calculated.

##### mRNA

For mRNA analysis, expression was initially quantified using HTSeq (v0.9.1), where the Read Count values for each gene were calculated as the original expression. These values were then standardized using FPKM. Differential expression genes (DEGs) was analyzed with DESeq (v1.38.3), using the following criteria:|log2FoldChange| > 1 and a significant P-value < 0.05. To visualize the differential expression, volcano plots were generated using the R language ggplot2 package. For enrichment analysis, all genes were mapped to terms in the Gene Ontology (GO) database, and the number of differentially enriched genes in each term was calculated. GO enrichment analysis was performed using topGO (v2.50.0), with significant enrichment determined by a P-value < 0.05. The enriched GO terms were identified to explore the main biological functions of DEGs. Additionally, Kyoto Encyclopedia of Genes and Genomes (KEGG) pathway enrichment analysis of the differential genes was carried out using ClusterProfiler (v4.6.0), focusing on pathways with a P-value < 0.05.

##### miRNA

For miRNA analysis, a reference genome index is built using Bowtie2 (v2.5.1), and the deduplicated clean reads are mapped to the reference genome using miRDeep2 (v2.0.0.8). Small RNA classification begins with annotation of unique reads using known miRNAs from the miRBase database, followed by the annotation of other non-coding RNAs. Sequences not annotated are predicted as new miRNAs using mireap (v0.2). The expression level of miRNAs is calculated based on the number of reads aligned to the mature miRNA. Differential expression analysis is performed with DESeq (v1.39.0), where miRNAs with|log2FoldChange| > 1 and P-value < 0.05 are considered differentially expressed. A volcano plot of the differentially expressed miRNAs (DEmiRNAs) is generated using the R language package ggplot2. Target gene prediction for DEmiRNAs is done using MiRanda (v3.3a), with the 3’ UTR sequence of mRNA as the target. Finally, GO and KEGG enrichment analyses are conducted on the target genes of the DEmiRNAs. GO enrichment is performed using topGO (v2.50.0), with a significance threshold of P-value < 0.05, and KEGG pathway analysis is carried out using clusterProfiler (v4.6.0), focusing on significantly enriched pathways with P-value < 0.05.

##### lncRNA

For lncRNA analysis, Stringtie (v2.2.1) software was used to assemble transcripts from HISAT2 (v2.1.0) alignment results, and candidate lncRNAs were screened based on splicing information and structural features. The coding potential of these candidate lncRNAs was predicted using three software tools (PLEK v1.2, CNCI v1.0, and Pfamscan v1.6.4). LncRNAs were confirmed when all three tools indicated non-coding RNA. Expression levels were analyzed using FPKM (fragments per kilobase of exon per million fragments mapped) to assess the distribution of lncRNA expression across samples, revealing that most lncRNAs were moderately expressed. Differential expression lncRNAs (DElncRNAs) was analyzed with DESeq (v1.38.3), identifying those with|log2FoldChange| > 1 and P-value < 0.05 as differentially expressed. Target gene prediction was carried out for both cis- and trans-acting lncRNAs. Cis-target genes were predicted by searching for protein-coding genes within 100KB upstream or downstream of the lncRNA, while trans-target genes were identified by calculating the expression correlation between lncRNA and mRNA using Pearson correlation or co-expression analysis. The interaction network between DElncRNAs and their target genes was visualized using the Igraph package. GO and KEGG enrichment analyses were performed on the target genes of DElncRNAs, with significant enrichment determined by P-value < 0.05. Finally, interaction analysis of lncRNA and miRNA was performed using MiRanda (v3.3a) and psRobot (v1.2) to predict potential miRNA target genes, exploring the ceRNA interactions involved in gene regulation.

##### circRNA

For circRNA analysis, 20 bp from both ends of unmapped reads from Tophat2 (2.0.14) alignment results were intercepted as anchor reads, and these were realigned to the genome using Bowtie2 (v2.5.1) for circRNA detection. CircRNAs were identified using find_circ (v1.0), and high-confidence circRNAs were screened. The expression levels of circRNAs were calculated using the Transcripts Per kilobase of exon model per Million mapped reads (TPM) method. Differential expression circRNAs (DEcircRNAs) analysis was performed using DESeq (v1.38.3), with transcripts having|log2FoldChange| > 1 and P-value < 0.05 considered differentially expressed. GO and KEGG enrichment analyses were conducted on the source genes of DEcircRNAs. GO enrichment was done with topGO (v2.50.0), focusing on significantly enriched terms with P-value < 0.05, and KEGG pathway enrichment was carried out with ClusterProfiler (v4.6.0), focusing on pathways with P-value < 0.05. Additionally, miRNA target genes in newly identified circRNAs were predicted using MiRanda (v3.3a) and psRobot (v1.2) software.

### Real-time reverse transcription-quantitative polymerase chain reaction (RT-qPCR) verification

Total RNA was extracted from 20 mg of embryo tissue, which was placed into a pre-chilled grinding tube, followed by the addition of 1 mL of RNA extraction reagent (G3013, Servicebio, Wuhan, China) and grinding beads. After pre-cooling on ice, the tissue was homogenized using a grinding instrument (KZ-5 F-3D, Servicebio, Wuhan, China) until no visible tissue chunks remained. The sample was then centrifuged at 4 °C, 12,000 rpm for 10 min, and the supernatant was collected. To this, 100 µL of chloroform substitute (G3014, Servicebio, Wuhan, China) was added, and the mixture was inverted for 15 s, allowed to stand for 3 min, and centrifuged at 4 °C, 12,000 rpm for 10 min. The supernatant (400 µL) was carefully transferred to a new tube and mixed with 550 µL of isopropanol (80109218, Sinopharm Chemical Reagent, Shanghai, China), then incubated at -20 °C for 15 min to precipitate the RNA. After centrifugation, the supernatant was discarded, and the precipitate was washed twice with 1 mL of 75% ethanol (10009218, Sinopharm Chemical Reagent, Shanghai, China). The RNA was then dissolved in RNA dissolving solution (G3029, Servicebio, Wuhan, China), and its concentration and purity were determined using a spectrophotometer (NanoDrop 2000, Thermo Scientific, Waltham, Massachusetts, USA).

For reverse transcription, a reaction system of 20 µL was prepared following the instructions of the reverse transcription kit (G3337, Servicebio, Wuhan, China), including 4 µL of 5×SweScript All-in-One SuperMix, 1 µL of gDNA Remover, 10 µL of total RNA, and 20 µL of RNase-free water (G4700). After mixing and centrifuging, the reaction tube was placed into a PCR machine (ETC811, Eastwin, Beijing, China), and the reverse transcription reaction was carried out with the following program: 5 min at 25 °C for pre-reaction, 30 min at 42 °C for reverse transcription, and 5 s at 85 °C to terminate the reaction. The resulting cDNA samples were used as templates for quantitative PCR.

Quantitative PCR was performed by mixing the cDNA with primers and SYBR Green Master Mix to prepare the reaction system. Three replicate reactions were set for each sample with the following components: 7.5 µL of 2×Universal Blue SYBR Green qPCR Master Mix (G3326, Servicebio, Wuhan, China), 1.5 µL of primers, 2 µL of cDNA, and 4 µL of RNase-free water, totaling 15 µL. The reaction plate was sealed, centrifuged, and placed into a quantitative PCR instrument (CFX Connect, Bio-Rad, California, USA). The amplification program included an initial denaturation at 95 °C for 30 s, followed by 40 cycles of denaturation at 95 °C for 15 s, and annealing/extension at 60 °C for 30 s. After the reaction, the Ct values for each well were recorded, with GAPDH or U6 used as internal controls. The relative expression levels were then calculated using the 2^−△△CT^ method. Primers were synthesized by Servicebio, Wuhan, China (Table 1).

**Table 1:** Primer sequences

Name	Forward Primer	Reverse Rimer
GAPDH	CCTCGTCCCGTAGACAAAATG	TGAGGTCAATGAAGGGGTCGT
ATG9B	GAGCAGGACTATGAACGGCTAG	GTCCAGGTTCTGGATGTGATGC
Cdkn1a	GGGACAAGAGGCCCAGTACT	CAATCTGCGCTTGGAGTGA
Foxo4	GGAATCCTGGGGGCTGTAAC	GACAGGTTGTGACGGATGGA
U6	CTCGCTTCGGCAGCACA	AACGCTTCACGAATTTGCGT
miR-146b-5p	CTCAACTGGTGTCGTGGAGTCGGCAATTCAGTTGAGAGCCTATG	ACACTCCAGCTGGGTGAGAACTGAATTCCA
miR-293-5p	CTCAACTGGTGTCGTGGAGTCGGCAATTCAGTTGAGCAAAATGT	ACACTCCAGCTGGGACTCAAACTGTGTGAC
miR-292a-5p	CTCAACTGGTGTCGTGGAGTCGGCAATTCAGTTGAGCAAAAGAG	ACACTCCAGCTGGGACTCAAACTGGGGGCT

### Statistical analysis

Statistical analysis was performed using SPSS 22.0 software. Data that followed a normal distribution were expressed as mean ± standard deviation (SD). For comparisons among multiple groups, one-way analysis of variance (ANOVA) was used when variances were equal, with post hoc pairwise comparisons conducted using the LSD test. When variances were unequal, the Kruskal-Wallis H test was applied. P value of < 0.05 was considered statistically significant.

## Results

### Acupuncture improves ovarian function in POR

**Fig. 2:**
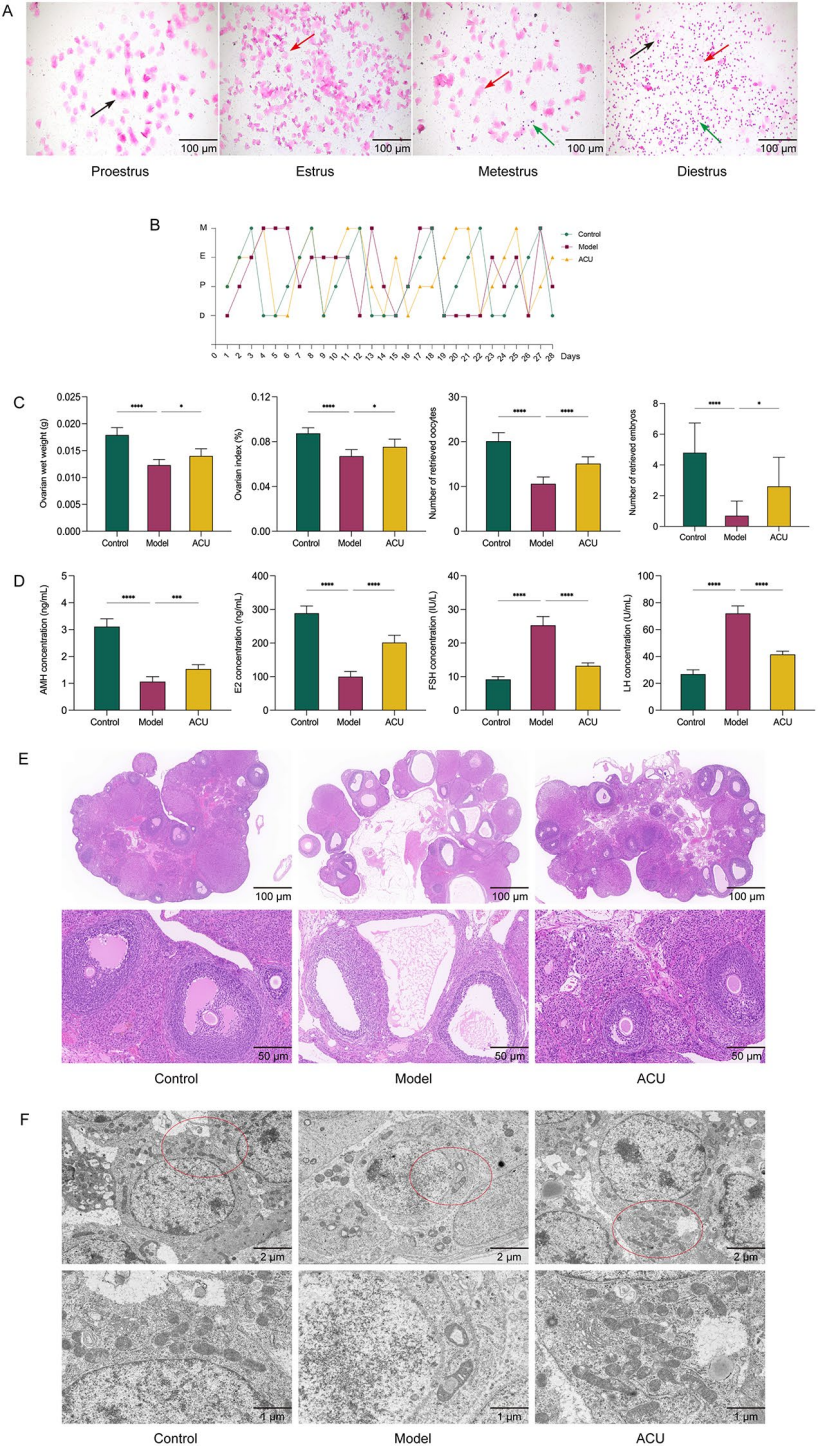
Acupuncture improves ovarian function in POR. (**A**) Comparison of vaginal exfoliated cell morphology across different estrous cycle stages. (**B**) Comparison of estrous cycle stages among groups. (**C**) Comparison of ovarian wet weight, ovarian index, and the number of retrieved oocytes and embryos among groups (*n* = 10). (**D**) Comparison of serum FSH, LH, AMH, and E2 concentrations among groups (*n* = 10). (**E**) Comparison of ovarian tissue morphology among groups. (**F**) Comparison of mitochondrial ultrastructure among groups. Black arrows indicate nucleated epithelial cells, red arrows indicate anucleated epithelial cells, and green arrows indicate leukocytes. Data are presented as mean ± SD. * *P* < 0.05, ** *P* < 0.01, *** *P* < 0.001, **** *P* < 0.0001

The estrous cycle is a key indicator of ovarian function, reflecting ovarian hormone secretion and follicular development [18]. A normal estrous cycle helps maintain ovarian health and reproductive capacity, while cycle abnormalities may indicate ovarian dysfunction. The mouse estrous cycle is divided into four stages: proestrus, characterized by numerous nucleated epithelial cells and some keratinized epithelial cells; estrus, dominated by fully keratinized, non-nucleated epithelial cells; metestrus, marked by leukocytes and occasional keratinized, non-nucleated epithelial cells; and diestrus, featuring a mix of leukocytes, nucleated epithelial cells, and non-nucleated epithelial cells (Fig. 2A). Prolonged estrous cycle or the absence of distinct cycle changes is considered a disruption of the estrous cycle. Compared with the control group, the model group mice exhibited significant estrous cycle disruption during both the modeling and treatment periods; after acupuncture treatment, the disruption was alleviated (Fig. 2B). The number of oocytes reflects the ovarian response to superovulation stimulation, while the number of embryos indicates the developmental potential of the retrieved oocytes and the efficiency of embryo formation. Compared with the control group, the model group showed a significant decrease in ovarian wet weight, ovarian index, number of oocytes, and number of embryos (*P* < 0.01); after acupuncture, these indicators increased to varying degrees (*P* < 0.01, *P* < 0.05) (Fig. 2C) (Supplementary 1). POR is typically accompanied by disordered serum hormone levels [8, 19], and our study found that, compared with the control group, the model group had decreased serum AMH and E2 levels (*P* < 0.01) and increased FSH and LH levels (*P* < 0.01); acupuncture reversed these trends (*P* < 0.01) (Fig. 2D).

In the context of POR, significant changes typically occur in the pathological structure of the ovary, leading to a decline in overall ovarian tissue function. HE staining revealed abnormal ovarian morphology in the model group, characterized by a marked reduction in the number of normal follicles at various stages and an increase in atretic follicles. After acupuncture treatment, both the quantity and morphology of normal follicles improved to varying degrees (Fig. 2E), indicating a restoration of ovarian function. Mitochondria are essential organelles for maintaining ovarian function, contributing to oocyte development and ovarian reserve through energy production, oxidative stress regulation, and involvement in apoptosis [20–21]. A reduction in mitochondrial quantity and abnormal morphology are closely associated with ovarian function decline. Therefore, TEM showed a decreased number of mitochondria in the ovarian tissue of the model group, with signs of incompleteness or swelling and cristae disruption. After acupuncture, the number of mitochondria increased, and mitochondrial structural damage was alleviated to varying degrees (Fig. 2F).

### Acupuncture improves apoptosis in ovarian cells of POR

**Fig. 3:**
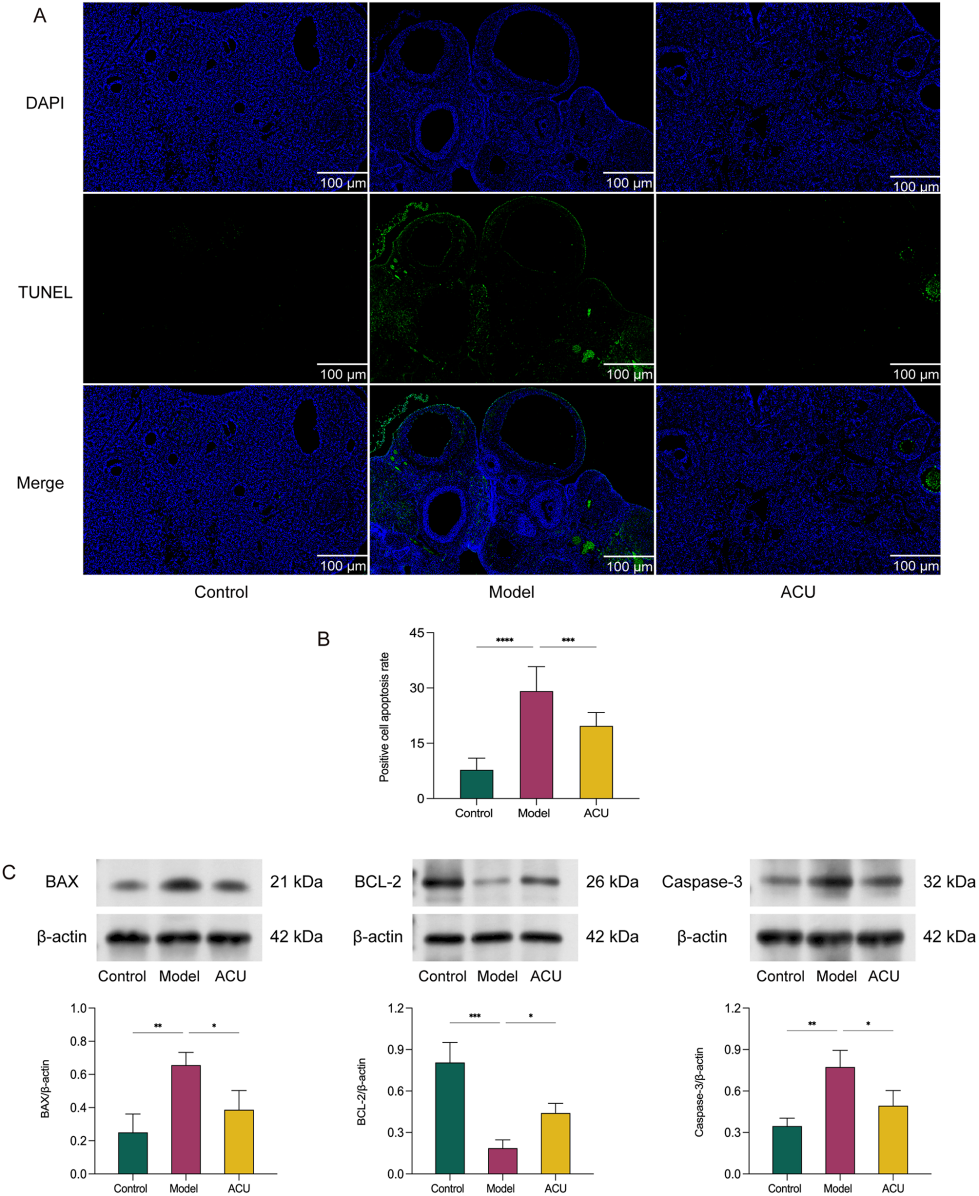
Acupuncture improves apoptosis in POR ovarian cells. (**A-B**) Comparison of positive cell apoptosis rates in ovaries among groups (*n* = 3). (**C**) Comparison of BAX, BCL-2, and Caspase-3 protein expression levels in testes among groups (*n* = 3). Data are presented as mean ± SD. * *P* < 0.05, ** *P* < 0.01, *** *P* < 0.001, **** *P* < 0.0001

POR is often accompanied by a reduced number of retrieved oocytes and decreased oocyte quality, which is closely linked to excessive apoptosis in oocytes [22]. The abnormal activation of apoptotic pathways accelerates ovarian function decline, leading to a dual reduction in both the quantity and quality of oocytes, thereby impacting fertility. Therefore, we observed apoptosis in ovarian cells using TUNEL staining. We found that the apoptosis level in the ovaries of model group mice was significantly higher compared to the control group (*P* < 0.01), while acupuncture markedly improved this condition (*P* < 0.01) (Fig. 3A-B). Additionally, we assessed the expression of apoptosis-related proteins BAX, BCL-2, and Caspase-3. Compared to the model group, the acupuncture group showed decreased expression levels of BAX and Caspase-3 and increased expression levels of BCL-2, indicating that acupuncture can alleviate apoptosis in POR (Fig. 3C) (Supplementary 6).

### The results of whole transcriptome analysis

#### Analysis of differentially expressed mRNA and ncRNA

**Fig. 4:**
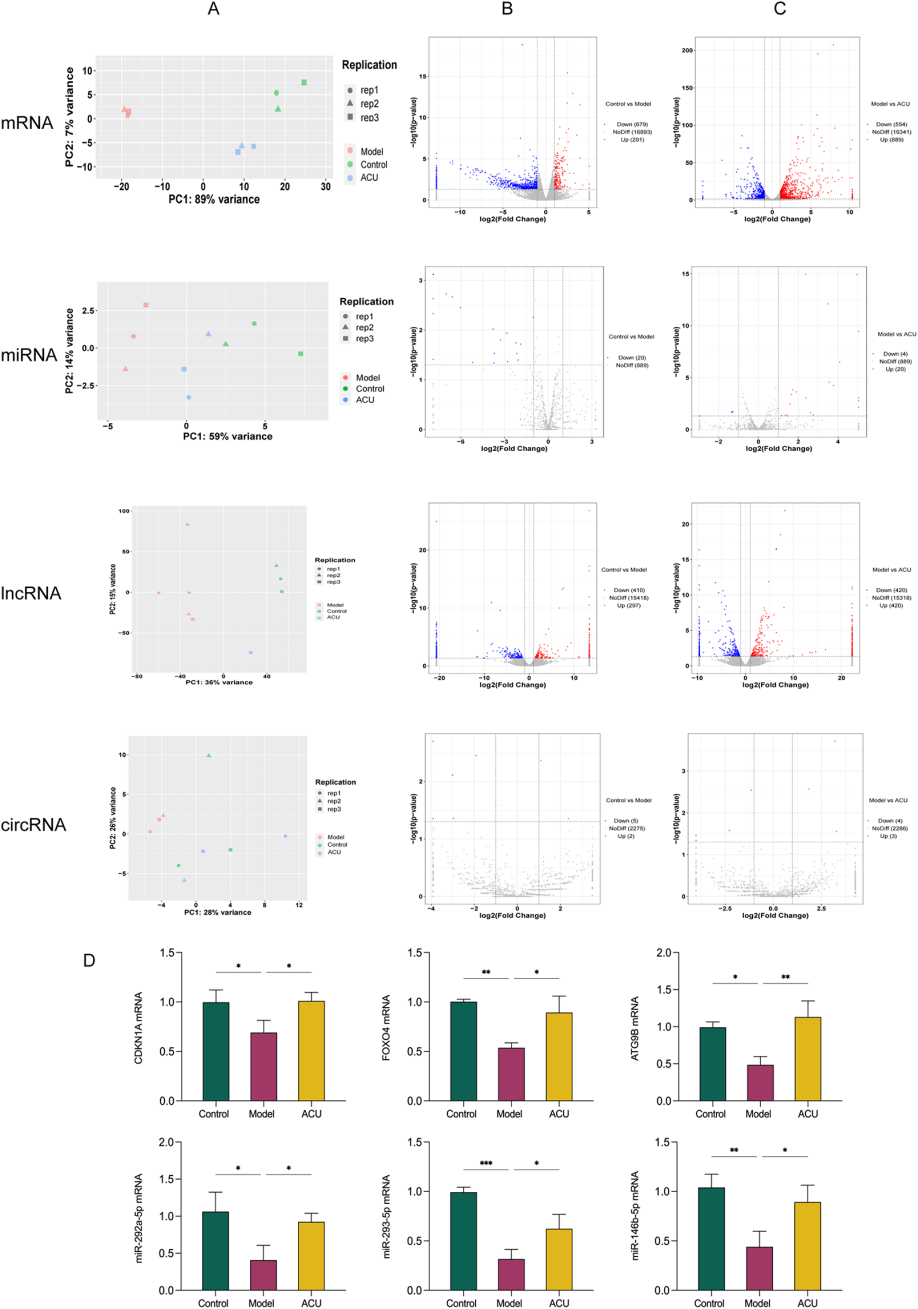
Analysis of DEmRNAs and DEncRNAs. (**A**) PCA correlation analysis. (**B**) Volcano plot of control group vs. model group. (**C**) Volcano plot of model group vs. ACU group. (**D**) DEmRNAs and DEncRNAs were validated by RT-qPCR (*n* = 3). Data are presented as mean ± SD. * *P* < 0.05, ** *P* < 0.01, *** *P* < 0.001, **** *P* < 0.0001. Note: In Figs. 4**B-C**, the two vertical dashed lines represent the threshold for fold change, while the horizontal dashed line represents the significance threshold. Red circles represent upregulated DEmRNA and DEncRNA, blue circles represent downregulated DEmRNA and DEncRNA, and gray circles represent mRNA and ncRNA with no statistically significant differences

The principal component analysis (PCA) of all identified mRNA and ncRNA (including miRNA, lncRNA, and circRNAs) showed significant differences between the groups (Fig. 4A). Based on the criteria of|log2FoldChange| > 1 and P-value < 0.05, we identified DEGs (Fig. 4B). Between the control group and the model group, 880 DEmRNAs (201 upregulated and 679 downregulated) were identified (Supplementary 2), along with 20 DEmiRNAs (0 upregulated and 20 downregulated) (Supplementary 3), 707 DElncRNAs (297 upregulated and 410 downregulated) (Supplementary 4), and 7 DEcircRNAs (2 upregulated and 5 downregulated) (Supplementary 5). Between the model group and the ACU group, 1443 DEmRNAs (889 upregulated and 554 downregulated), 24 DEmiRNAs (20 upregulated and 4 downregulated), 840 DElncRNAs (420 upregulated and 420 downregulated), and 7 DEcircRNAs (3 upregulated and 4 downregulated) were identified. Additionally, to compare the significant differences in gene expression between the control vs. model group and the model vs. ACU group, volcano plots were generated to visualize the DEGs (Fig. 4C). To validate the accuracy of the whole-transcriptome analysis results, we randomly selected six differentially expressed mRNAs and ncRNAs for qRT-PCR analysis, including three mRNAs (CDKN1A, FOXO4, ATG9B) and three miRNAs (miR-292a-5p, miR-293-5p, miR-146b-5p). We found that the qRT-PCR results were consistent with the expression trends observed in the DEGs, indicating that the subsequent analysis results are reliable (Fig. 4D).

#### Enrichment analysis of GO and KEGG

**Fig. 5:**
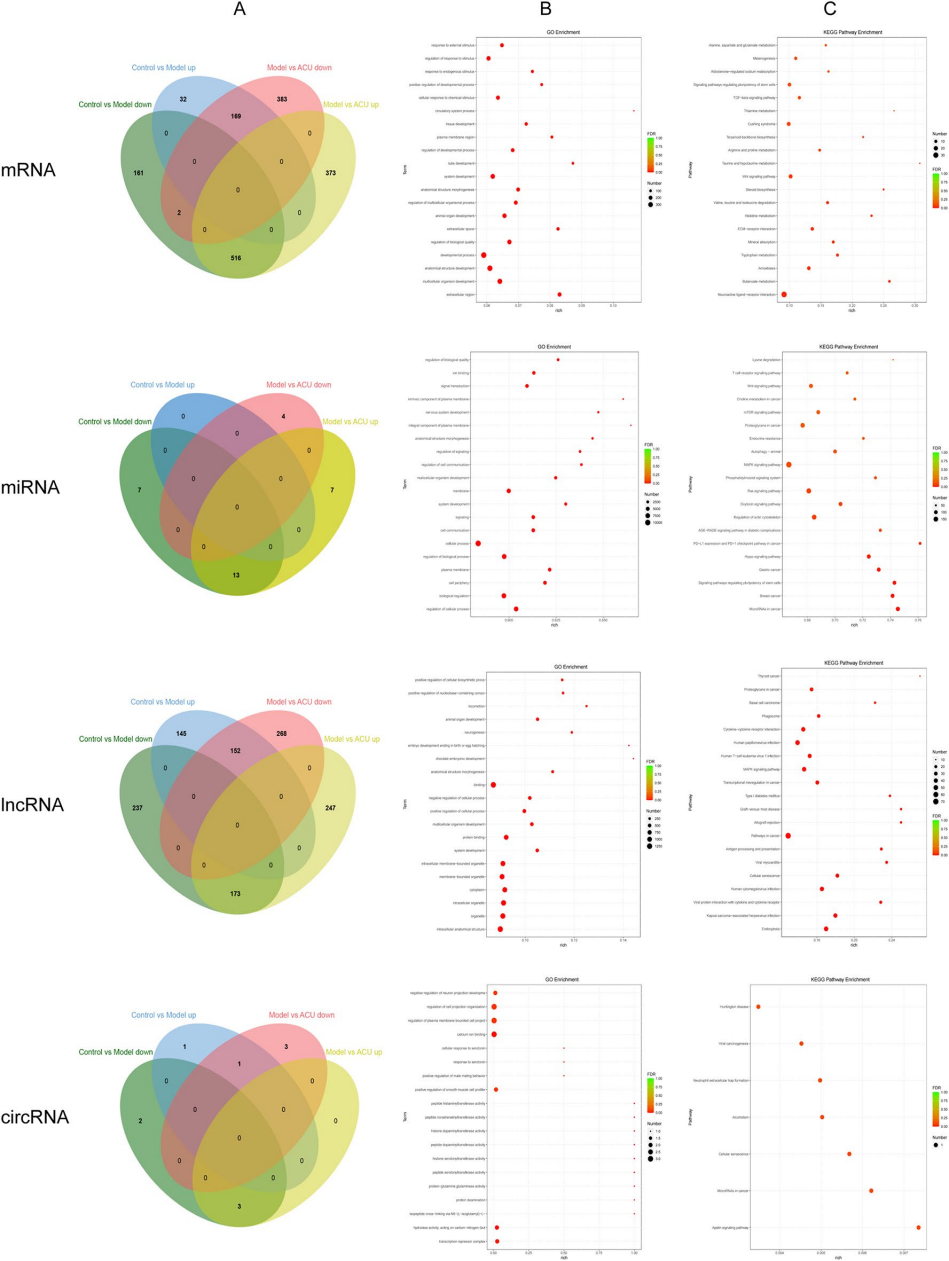
GO and KEGG enrichment analysis. (**A**) Venn diagram of intersecting DEmRNAs and DEncRNAs with reversal trends. (**B**) GO analysis. (**C**) KEGG enrichment analysis

This study aimed to explore the mechanisms by which acupuncture affects early embryo development in POR mice following IVF-ET. Therefore, we primarily focused on the common DE mRNAs and DE ncRNAs between the control vs. model group and the model vs. ACU group (Fig. 5A). We identified 169 DEmRNAs, 0 DEmiRNAs, 152 DElncRNAs, and 1 DEcircRNA that were upregulated in the model group and downregulated after acupuncture treatment. Additionally, 516 DEmRNAs, 13 DEmiRNAs, 173 DElncRNAs, and 3 DE circRNAs were downregulated in the model group and upregulated after acupuncture treatment. As these DEmRNAs and DEncRNAs showed significant differential expression changes before and after modeling and treatment, they may serve as potential targets for acupuncture to improve early embryo development and will be used for subsequent functional enrichment analysis. GO analysis revealed that DEmRNAs are primarily involved in processes such as response to external stimulus, regulation of response to stimulus, and response to endogenous stimulus. DEmiRNAs are mainly involved in regulation of biological quality, ion binding, and signal transduction. DElncRNAs are primarily associated with positive regulation of cellular biosynthetic processes, positive regulation of nucleobase-containing compounds, and locomotion. DEcircRNAs are mainly involved in negative regulation of neuron projection development, regulation of cell projection organization, and regulation of plasma membrane-bounded cell projections (Fig. 5B). KEGG enrichment analysis showed that DEmRNAs are mainly associated with signaling pathways such as the TGF-beta signaling pathway and Wnt signaling pathway. DEmiRNAs are involved in pathways including the Wnt signaling pathway, mTOR signaling pathway, and MAPK signaling pathway. DElncRNAs are linked to pathways such as the MAPK signaling pathway, and DEcircRNAs are associated with the Apelin signaling pathway (Fig. 5C).

#### Construction of the lncRNA/circRNA-miRNA-mRNA network

**Fig. 6:**
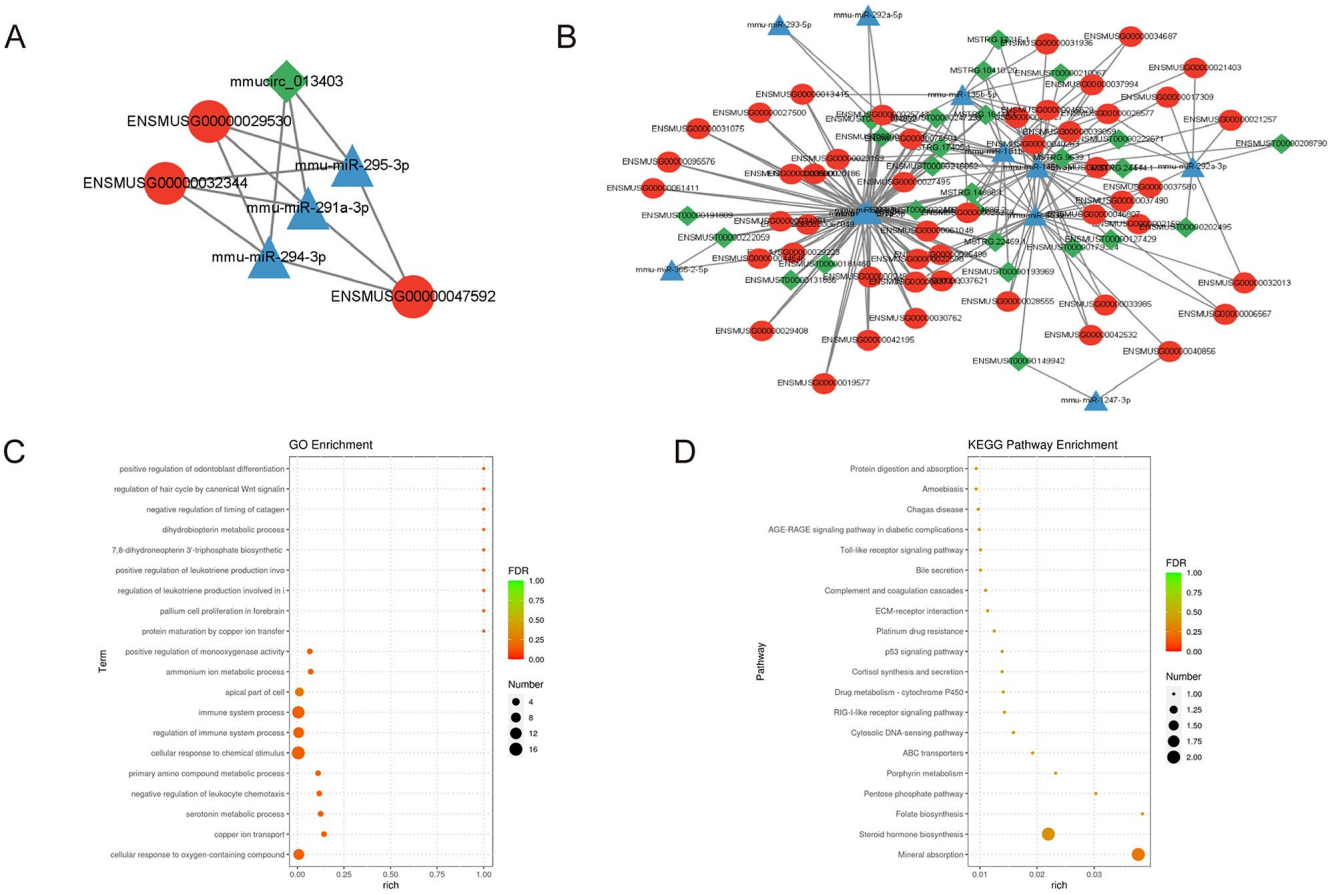
The network of lncRNA/circRNA-miRNA-mRNA. (**A**) The interaction network of circRNA–miRNA–mRNA. (**B**) The interaction network of lncRNA–miRNA–mRNA. (**C**) GO analysis. (**D**) KEGG enrichment analysis

Constructing an ncRNA-mRNA regulatory network helps to reveal the key role of ceRNAs in regulating gene expression, aiding our understanding of how lncRNAs and circRNAs influence mRNA stability and translation through competitive binding with miRNAs [23]. Based on the previously identified DEmRNAs and DEncRNAs, we screened for lncRNA/circRNA-miRNA-mRNA relationships with ceRNA associations (by selecting results with sensitive correlations greater than 0.3) and visualized the network using Cytoscape (Fig. 6A-B). We found that miR-291a-3p, miR-294-3p, and miR-295-3p may serve as core targets within the ceRNA network, participating in the regulation of mRNAs by lncRNAs/circRNAs. Additionally, for these lncRNA/circRNA-miRNA-mRNA relationships with ceRNA associations, we performed GO and KEGG enrichment analyses using hypergeometric testing on the mRNA (target gene set) (Fig. 6C-D), revealing their close connection to pathways such as the Toll-like receptor signaling pathway and the p53 signaling pathway.

## Discussion

POR refers to inadequate ovarian response to gonadotropin stimulation during ovulation induction treatment, typically characterized by a low number of retrieved follicles and poor oocyte quality. POR is commonly observed in women with diminished ovarian reserve, especially in older patients. Due to the reduction in follicle count and oocyte quality, POR significantly decreases the likelihood of successful conception and poses a significant challenge for reproductive treatments [1–4]. This study found that, compared to the control group, the model group mice exhibited reductions in the number of retrieved oocytes, ovarian wet weight, and ovarian index, along with a decrease in the number of normal follicles at various stages and an increase in atretic follicles. Serum levels of FSH and LH were elevated, while AMH and E2 levels were decreased. Mitochondria play a crucial role in ovarian function by providing energy for oocyte development and maturation, and regulating apoptosis and oxidative stress. Mitochondrial dysfunction can lead to decreased ovarian function and poor oocyte quality, thereby affecting fertility [21, 24]. The study observed a reduction in the number of mitochondria and abnormalities in the morphology and internal structure of mitochondria in the ovarian tissues of the model group, indicating successful establishment of the POR mouse model. Acupuncture is an important component of TCM, and several clinical studies have shown its positive effects in treating POR and other ovarian disorders. Acupuncture can enhance ovarian function by stimulating specific acupuncture points to improve qi and blood circulation and increase ovarian blood supply. Additionally, acupuncture can regulate hormone levels, improve endocrine balance, and reduce inflammation in the ovaries and surrounding tissues [13, 14, 15, 25–26]. This study used acupuncture at the points “Tai Chong” (LR3), “San Yin Jiao” (SP6), and “Guan Yuan” (CV4) for treatment. Acupuncture at “Tai Chong” (LR3) helps regulate liver function and improve qi and blood circulation, thereby enhancing ovarian blood supply. Acupuncture at “San Yin Jiao” (SP6) can regulate spleen, stomach, liver, and kidney functions, positively affecting the menstrual cycle and ovarian endocrine function. Acupuncture at “Guan Yuan” (CV4) has a strong kidney-replenishing effect, enhancing physical vitality and improving ovarian function. The combination of these points helps to improve overall ovarian function and alleviate POR symptoms. The study found that after acupuncture treatment, the number of normal follicles at various stages increased in the mice ovaries, with improvements in the number of retrieved oocytes, ovarian wet weight, and ovarian index. Serum hormone levels were corrected, and mitochondrial quantity and morphology improved, suggesting that acupuncture can effectively ameliorate reduced ovarian response and play a positive role in ovulation induction and ovarian protection.

Apoptosis is a crucial aspect of ovarian health, playing a significant role in regulating follicle development, maintaining the quality and quantity of ovarian follicles, and influencing ovarian aging [27–28]. Proper apoptotic mechanisms ensure the elimination of immature or suboptimal follicles, thereby maintaining the developmental potential of remaining follicles. However, in cases of POR, apoptotic regulation may become dysregulated. Notably, with advancing age, increased natural apoptosis in the ovary can exacerbate follicle depletion, thereby affecting female fertility. Additionally, hormone levels and pathological conditions can influence apoptotic regulation and ovarian function. Key molecules in apoptosis regulation, such as BAX, BCL-2, and Caspase-3, are critical for maintaining ovarian function [29]. Our study found that in the model group, ovarian granulosa cell apoptosis was increased, with elevated levels of BAX and Caspase-3 and reduced levels of BCL-2. Acupuncture reversed these trends, suggesting that acupuncture may improve ovarian function by inhibiting excessive apoptosis, thus helping to maintain follicle quantity within the ovary.

IVF-ET represents a significant advancement in assisted reproductive technology, offering hope for individuals with infertility. Despite ongoing improvements in this technique, clinical pregnancy rates remain suboptimal, and concerns regarding offspring health continue to arise. Clinical pregnancy rates are influenced by several factors, including embryo quality, endometrial receptivity, maternal health, and laboratory conditions. Even the transfer of high-quality embryos faces challenges in achieving and maintaining a full-term pregnancy. Furthermore, potential risks to offspring from IVF-ET, such as preterm birth, low birth weight, birth defects, and neurodevelopmental disorders, have been extensively reported [30–32]. These risks may be associated with supra-physiological hormone levels, embryonic manipulation, and cryopreservation techniques, as well as genetic and epigenetic alterations. Therefore, assessing early embryo quality during IVF-ET is crucial. The occurrence of POR makes achieving pregnancy via IVF-ET more challenging. However, increasing evidence suggests that acupuncture may positively impact IVF-ET outcomes in POR patients [15, 33]. Acupuncture may regulate the hypothalamic-pituitary-ovarian (HPO) axis, improve hormonal balance, and enhance ovarian responsiveness to ovulation-inducing drugs, effectively promoting follicle development and oocyte maturation, thereby improving egg yield and quality. Additionally, acupuncture can enhance local ovarian blood circulation, improve oxygen and nutrient delivery, optimize follicular fluid environment, reduce inflammation, and boost antioxidant capacity, all of which positively affect embryo development. These mechanisms may not only improve embryo quality and increase the number of viable embryos but also enhance endometrial receptivity, creating a more favorable environment for embryo implantation. However, current research primarily focuses on the effects of acupuncture on the maternal body, while in-depth exploration of the embryos formed from oocytes treated with acupuncture and subjected to IVF-ET remains limited. Embryo development begins with the oocyte, and the quality of the oocyte plays a crucial role in the progression and potential of embryo development. Our study found that after acupuncture treatment in donor mice, a series of positive changes occurred, including stabilization of serum hormone levels, improved ovarian function, and reduced apoptosis. These changes created an optimal environment for oocyte growth and maturation, significantly enhancing both the quality and quantity of oocytes. As a result, the oocytes carried more favorable substances and signals for embryo development, which were transmitted to the embryo level, influencing its developmental potential and quality. By using whole-transcriptome sequencing, we performed gene expression analysis on embryos formed from oocytes derived from acupuncture-treated donors. This approach allowed us to comprehensively capture changes in embryo gene expression, revealing how acupuncture may influence embryo development at the molecular level by optimizing oocyte quality. We primarily focused on the 685 DEmRNAs, 13 DEmiRNAs, 325 DElncRNAs, and 4 DEcircRNAs identified between the control vs. model group and model vs. ACU group, showing a trend of reversal. Specifically, 169 DEmRNAs, 0 DEmiRNAs, 152 DElncRNAs, and 1 DEcircRNA were upregulated in the model group but downregulated after acupuncture treatment. Conversely, 516 DEmRNAs, 13 DEmiRNAs, 173 DElncRNAs, and 3 DEcircRNAs were downregulated in the model group but upregulated after acupuncture treatment. Many of these DEmRNAs and DEmiRNAs have been shown to be related to embryo development and are consistent with the trends observed in our experiment. However, most of the DElncRNAs and DEcircRNAs are unknown, largely due to the scarcity of related research. miR-146b-5p plays a significant role in embryo development and intercellular communication at the maternal-fetal interface. Research shows that miR-146b-5p is highly expressed in pre-blastocyst embryos and their secreted extracellular vesicles (EVs), inhibiting genes associated with embryonic developmental apoptosis [34–35]. Additionally, in recurrent spontaneous abortion (RSA), miR-146b-5p is transported to trophoblast cells via EVs secreted by M1 macrophages, inhibiting their migration and invasion abilities, further exacerbating embryo resorption [36]. CDKN1A (p21) is an important molecule in regulating embryo development, primarily controlling the cell cycle process by inhibiting CDKs. It is involved in regulating cell proliferation, differentiation, and apoptosis during early embryo development. In response to DNA damage signals, CDKN1A also promotes DNA repair or induces apoptosis in damaged cells, thus maintaining genomic integrity during embryonic development [37–38]. ATG9B is a crucial autophagy protein that plays a key role in embryonic development. It helps in the formation and expansion of autophagosomes, regulates the clearance and recycling of intracellular materials, and supports normal cell proliferation and differentiation during embryogenesis. ATG9B also helps cells cope with developmental stress, maintaining healthy embryo development [39–40]. Dysfunctional ATG9B can lead to insufficient autophagy, affecting embryo development. In summary, this suggests that acupuncture in POR maternal mice may help regulate changes in these key DEmRNAs and DEncRNAs in the offspring embryos, supporting the formation of multiple tissues and organs in early embryos and reducing the risk of gene mutation.

Constructing ncRNA-mRNA networks allows for a deeper understanding of how lncRNAs and circRNAs influence mRNA stability and translation through competitive binding with miRNAs, thereby impacting cellular functions and disease progression [41]. This also offers potential new targets and strategies for early disease diagnosis and treatment. From the ceRNA network, it can be observed that miR-291a-3p, miR-294-3p, and miR-295-3p can simultaneously connect to 5 lncRNAs and 1 circRNA, targeting multiple distinct mRNAs. This suggests that these miRNAs may be key regulatory molecules in the mechanism by which acupuncture improves embryo quality. miR-291a-3p, miR-294-3p, and miR-295-3p all belong to the miR-290 family, a mouse-specific miRNA family that is highly expressed in embryonic stem cells, primordial germ cells, and early embryonic development. These miRNAs play roles in maintaining stem cell self-renewal, promoting cell proliferation, inhibiting apoptosis, and enhancing DNA repair. By targeting and inhibiting cell cycle negative regulators and apoptosis-related genes, members of the miR-290 family are crucial in maintaining the undifferentiated state and rapid proliferation of embryonic stem cells [42–44]. Additionally, KEGG analysis revealed that the ceRNA network mainly affects the Toll-like receptor signaling pathway and the p53 signaling pathway. TLRs are crucial receptors in the innate immune system, initiating immune responses by recognizing pathogen-associated molecular patterns (PAMPs) and danger-associated molecular patterns (DAMPs). During embryonic development, the TLR signaling pathway not only plays a role in maintaining maternal immune balance and protecting the embryo from infections but also potentially influences normal embryonic development by regulating apoptosis, inflammatory responses, and tissue repair [45–47]. p53, known as the “guardian of the genome,” primarily functions to regulate the cell cycle, promote apoptosis, maintain genomic stability, and participate in DNA damage repair. In the early stages of embryonic development, the p53 signaling pathway ensures the proper division and differentiation of zygotes and embryonic cells through precise regulation, preventing cells with DNA damage or mutations from continuing to proliferate [48–50]. If p53 function is impaired or mutated, it may lead to abnormal embryonic development or even early embryonic death.

This study investigated the effects of acupuncture on POR mice and preliminarily elucidated its potential mechanisms through embryonic transcriptome analysis. However, several limitations need to be addressed in future research. First, the study primarily relies on embryonic transcriptome data to explore the mechanisms of acupuncture treatment. Although improvements in embryo quality may indirectly reflect enhanced maternal ovarian function, the lack of synchronized transcriptomic analysis of ovarian tissues prevents the direct establishment of a molecular link between acupuncture-induced ovarian regulation and embryonic development, limiting the depth of mechanistic interpretation. Second, the use of pseudopregnant mice as embryo recipients may introduce additional physiological variables, potentially affecting the interpretation of embryonic transcriptome data. Although strict controls were implemented on recipient mice, including age, weight, and health status, the potential systematic bias introduced by pseudopregnancy remains to be further validated. To address these limitations, future studies will incorporate simultaneous transcriptomic analysis of both maternal ovarian tissues and embryos to compare co-expression patterns of ovarian function-related genes and key embryonic developmental genes, thereby clarifying whether acupuncture improves embryo quality by modulating the ovarian microenvironment. Additionally, we will integrate multi-omics approaches, including metabolomics and epigenomics, to comprehensively investigate the maternal-embryo crosstalk underlying acupuncture-induced reproductive benefits. Furthermore, the effects of recipient selection will be assessed by comparing embryonic transcriptomes from pseudopregnant and non-pseudopregnant recipients or utilizing autologous embryo transfer, ensuring a more precise evaluation of the direct regulatory effects of acupuncture on embryonic development.

## Conclusion

In conclusion, we identified the expression profiles of DEmRNAs, DEmiRNAs, DElncRNAs, and DEcircRNAs in early embryos from POR mice following acupuncture, based on whole-transcriptome sequencing. By constructing a circ/lncRNA-miRNA-mRNA network, we discovered that core targets such as miR-291a-3p, miR-294-3p, and miR-295-3p may influence multiple pathways, including the Toll-like receptor signaling pathway and p53 signaling pathway, potentially regulating embryonic development. However, further studies are needed to explore these mechanisms in more detail.

## Electronic supplementary material

Below is the link to the electronic supplementary material.

Supplementary Material 1

Supplementary Material 2

Supplementary Material 3

Supplementary Material 4

Supplementary Material 5

Supplementary Material 6

## Data Availability

All relevant data are within the paper and its Supplementary Materials. The Whole-transcriptomic raw data have been deposited in the NCBI SRA database (https://www.ncbi.nlm.nih.gov/sra) with accession number PRJNA1162234.
