# Supplementary figures and images for: Whole-transcriptome sequencing reveals the effects of acupuncture on early embryos post-IVF-ET in poor ovarian response
Source: J Ovarian Res. 2025 Apr 30;18:91. doi: 10.1186/s13048-025-01682-7 (PMC12044900; doi:10.1186/s13048-025-01682-7)

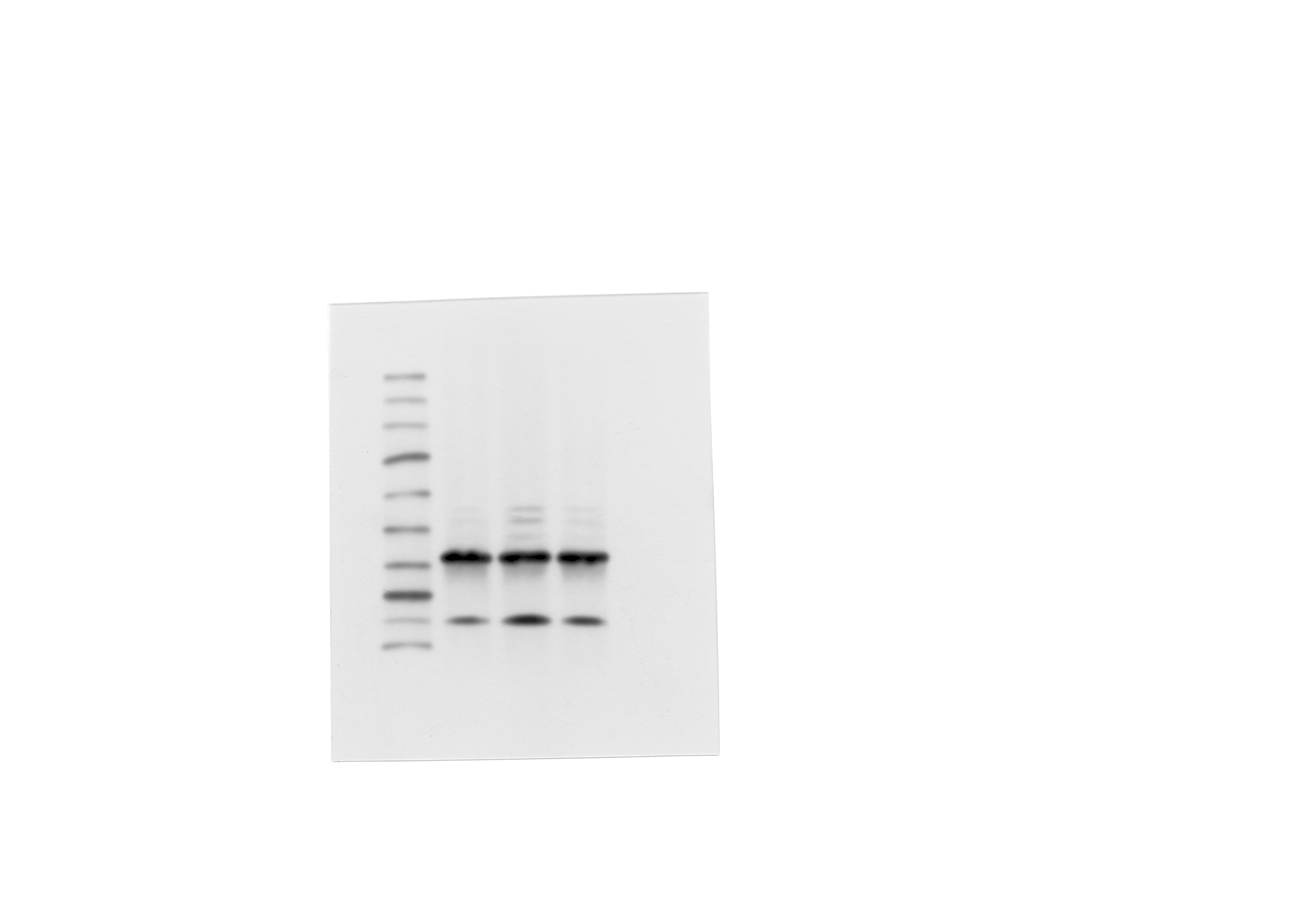

Supplement: Supplementary file 6 — Supplementary Material 6 [file 13048_2025_1682_MOESM6_ESM.zip › BAX.tif]

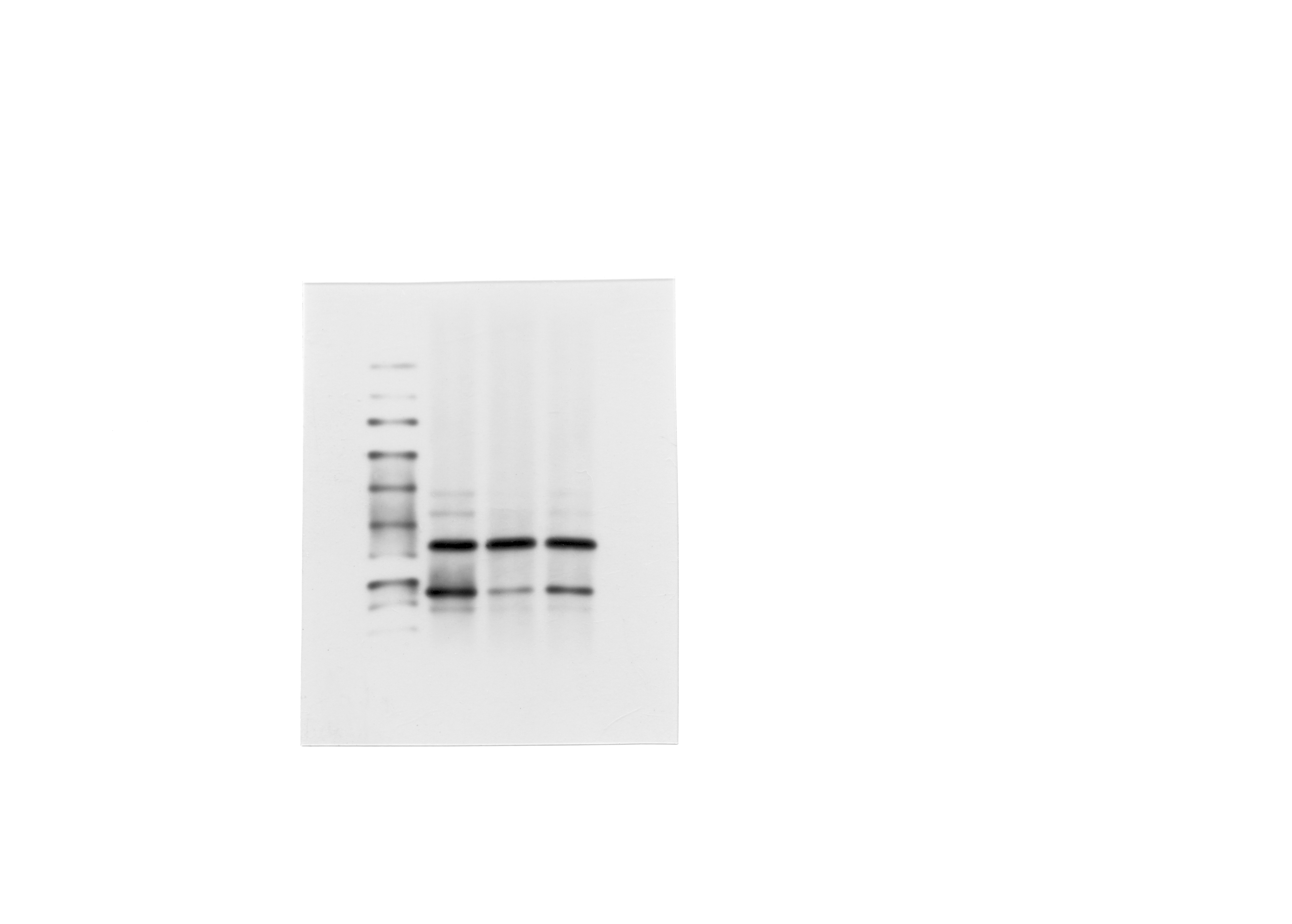

Supplement: Supplementary file 6 — Supplementary Material 6 [file 13048_2025_1682_MOESM6_ESM.zip › BCL-2.tif]

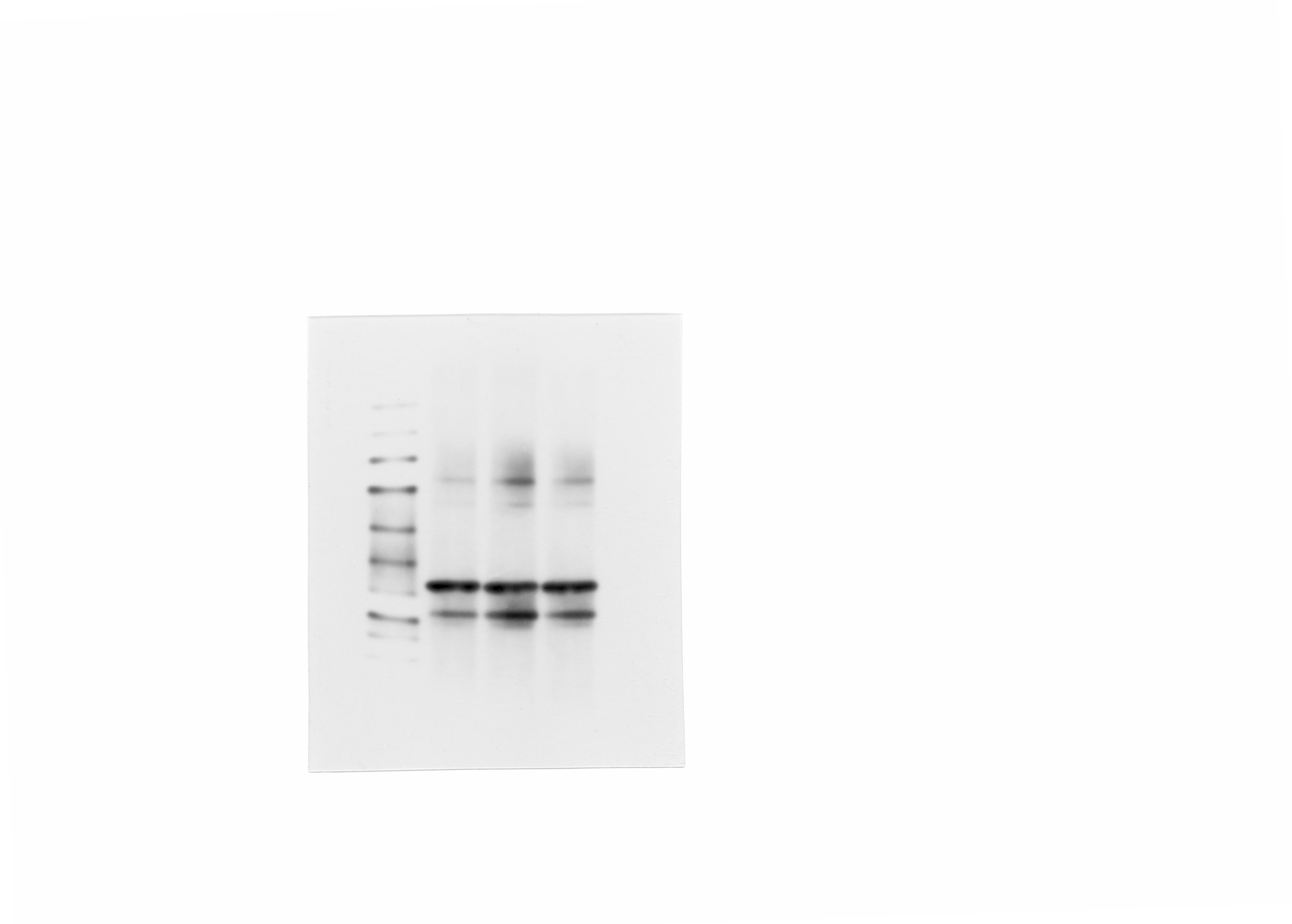

Supplement: Supplementary file 6 — Supplementary Material 6 [file 13048_2025_1682_MOESM6_ESM.zip › Caspase-3.tif]
